# A Novel Pathogenic Large Duplication in EXT1 Identified in a Family with Multiple Osteochondromas

**DOI:** 10.3390/genes15091169

**Published:** 2024-09-05

**Authors:** Isabella Bartolotti, Klaudia Sobul, Serena Corsini, Davide Scognamiglio, Alice Moroni, Maria Gnoli, Luca Sangiorgi, Elena Pedrini

**Affiliations:** Department of Rare Skeletal Disorders, IRCCS Istituto Ortopedico Rizzoli, 40131 Bologna, Italyserena.corsini@ior.it (S.C.); davide.scognamiglio@ior.it (D.S.);

**Keywords:** multiple osteochondromas, EXT1, large duplication

## Abstract

Multiple osteochondromas (MO) is an autosomal dominant disorder and the most common genetic skeletal dysplasia, characterized by the growth of bone outgrowths capped by cartilage, called osteochondromas. Most MO cases are caused by mutations in the exostosin-1 (*EXT1*) and exostosin-2 (*EXT2*) genes. Only 5% of MO-causative variants are represented by single or multiple exon deletions; to date, no pathogenic large duplication has been described in the literature. In the present study, we describe the novel in-tandem intragenic duplication c.(1128_1202)_(1284+29_1344)dup involving exon 4 of *EXT1* (NM_000127.2), detected in a three-generation family with MO. The variant has been detected by MLPA (multiplex ligation-dependent probe amplification) and then confirmed with qPCR (quantitative PCR). Our finding expands the spectrum of MO-causing variants describing a pathogenic large duplication, underlying the importance of quantitative analysis in patients with negative sequencing.

## 1. Introduction

Multiple osteochondromas (MO, MIM#133700, #133701) is an autosomal dominant hereditary disease, characterized by the formation of benign cartilage-capped bony outgrowths called osteochondromas (OCs) [1]. Osteochondromas, also named exostoses, most frequently develop from the juxta-epiphyseal region of long bones but can also occur in the diaphyseal area. Flat bones, vertebrae, and ribs may also be, but rarely are, involved. MO is the most common genetic skeletal dysplasia, with an underestimated prevalence of 1:50.000 in the Western population [2]. OCs are rarely present at birth: their proliferation and growth occur from early childhood until puberty at growth plate closure [3]. During adulthood, patients should undergo long-term follow-up; in fact, although OCs stop growing, in 0.5–2% of cases, they may undergo malignant transformation into secondary peripheral chondrosarcoma [4]. Most MO cases (70–95%, depending on the study) are caused by loss-of-function mutations in two genes: exostosin-1 (*EXT1*) located in 8q24.11- q24.13 (MIM 608177) and exostosin-2 (*EXT2*) sited in 11p12-q11 (MIM 608210) [5,6]. Both genes encode for glycosyl-transferases that form a Golgi-resident heterocomplex responsible for the elongation of the heparan sulfate (HS) chains of matrix proteoglycans [7,8,9]: the reduction in or shortening of HS chains, due to *EXT* mutations, could cause negative effects on chondrocyte proliferation and differentiation [10]. *EXT1*/*EXT2* mutations are detected via sequencing analyses of the coding regions, including the exon–intron boundaries, and deletion/duplication analyses using multiplex ligation-dependent probe amplification (MLPA) [7]: *EXT1* is mutated in 56–78% of Caucasian MO families, whereas *EXT2* in 21–44% [4,6]. Despite some discrepancies in the Asian and Latin American population, data obtained from the Human Gene Mutation Database (HGMD) “https://www.hgmd.cf.ac.uk/ac/index.php (accessed on 28 August 2024)” and the Multiple Osteochondromas Mutation Database “https://databases.lovd.nl/shared/genes/EXT1, https://databases.lovd.nl/shared/genes/EXT2 (accessed on 28 August 2024)” [4] evidence that the number of pathogenic variants in *EXT1* is higher than that of *EXT2* [10]. The majority of *EXT1* and *EXT2* alterations (77–80%) are inactivating point mutations: nonsense, small indels, and splice-site variants that cause inactivation of the gene, usually ending in a premature termination of EXT protein translation [4]. About 5% of MO-causative variants are represented by one or multiple exon deletions (including whole gene deletions); of note, no intragenic large duplication has been described to date as being pathogenic in the scientific literature. In the present study, a novel in-tandem intragenic duplication of *EXT1* exon 4 is described as the disease-causative variant in a three-generation family with MO.

## 2. Materials and Methods

### 2.1. Subjects of This Study

**Figure 1:**
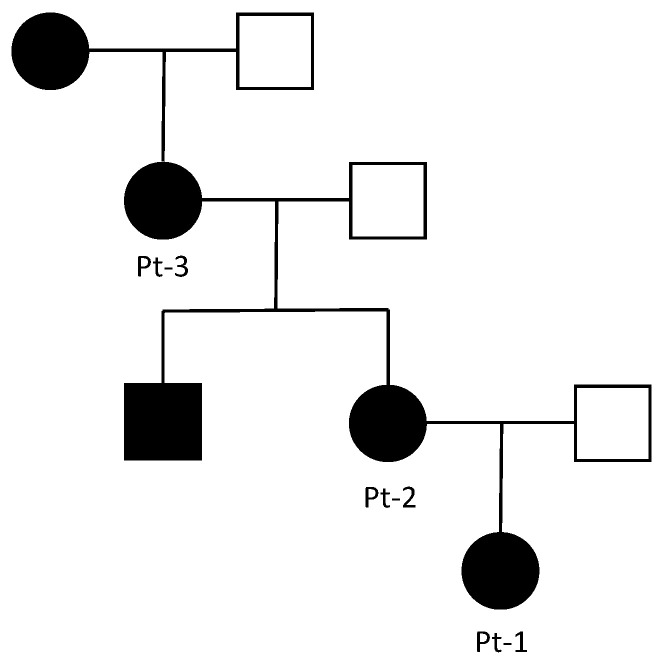
Family tree. All the reported affected relatives are represented in black. Pt: patient.

All subjects gave their informed consent to undergo diagnostic genetic tests as part of routine health care assessments. The subjects of this study belong to a three-generation Italian family affected by MO. The proband (Pt-1) is a 13-year-old girl with a family history for MO. She has a 41-year-old mother (Pt-2) and a 64-year-old grandmother (Pt-3), both affected by MO (Figure 1). Other affected family members were reported to the anamnesis, as described in Figure 1.

### 2.2. DNA Extraction

The genomic DNA of all patients, including an unaffected subject as a negative control (WT), was extracted from whole peripheral blood with Maxwell^®^ CSC 48 (Promega) through the Maxwell^®^ CSC Whole Blood DNA Kit (Promega), according to the manufacturer’s protocols. The extracted DNA was quantified through a Lunatic (Unchained Labs, Pleasanton, CA, USA) spectrophotometer to ensure proper concentrations and quality.

### 2.3. NGS Analysis

DNA samples were first screened for *EXT1* (NM_000127.2) and *EXT2* (NM_207122.1) point mutations, using a Next Generation Sequencing (NGS) IonTorrent platform (Thermo Fisher Scientific, Waltham, MA, USA). According to the manufacturer’s protocols, the libraries were obtained via Ion Ampliseq Library Kit Plus (Thermo Fisher Scientific) using a targeted panel (Thermo Fisher Scientific) covering all the coding exons of *EXT1* and *EXT2* genes, also including the intronic flanking regions. Different samples were barcoded using Ion Xpress Barcode Adapters (Thermo Fisher Scientific) and charged on a 520 chip (Thermo Fisher Scientific) via Ion Chef (Thermo Fisher Scientific), before the sequencing run on the Ion Gene studio S5 (Thermo Fisher Scientific). Sequencing data were analyzed using the Ion Torrent Suite Software (Thermo Fisher Scientific) and the SEQNEXT application (JSI Medical Systems GmbH, Ettenheim, Germany).

### 2.4. MLPA Analysis

To assess the presence of large deletions/duplications in *EXT1* and *EXT2* genes, a multiplex ligation-dependent probe amplification (MLPA) analysis was performed using a SALSA^®^ MLPA Reagent Kit and Probemix P215 EXT (MRC Holland, Amsterdam, The Netherlands) according to the supplier’s instructions. The MLPA assay was performed on a Veriti Thermal Cycler (Applied Biosystems, Waltham, MA, USA) during the incubation phases; then, fragments were run through a Genetic Analyzer 3500 XL (Applied Biosystems). Coffalyser.Net™ Software (MRC Holland) was used for the analysis of fragments. The MLPA results of each individual probe are expressed using the dosage quotient (DQ) as follow: 0.80 < DQ < 1.20 is related to a normal copy number status, 0.40 < DQ < 0.65 to a heterozygous deletion, and 1.30 < DQ < 1.65 to a heterozygous duplication. Any detected copy number variant (CNV) is classified following the American College of Medical Genetics and Genomics (ACMG) criteria [11].

### 2.5. qPCR Confirmation

The MLPA results were confirmed using the quantitative PCR (qPCR) technique. The assay was executed using RT2 SybrGreen qPCR Master Mix (Qiagen) and specific primers for *EXT1* exons 3, 4, and 5 to obtain a final concentration of 400 nM (primer sequences are reported in Supplementary Materials, Table S1). The *ACTB* gene was chosen as a reference. Rotor-Gene Q (Qiagen, Hilden, Germany) was used for the thermic protocol. Rotor-Gene Q Series Software (Qiagen; Software Version 2.0.2) was used for the analysis of the results. A Delta Delta CT Relative Quantitation Analysis was applied. qPCR results are expressed using the Relative Concentration (RC) as follow: 0.80 < RC < 1.20 is related to a normal copy number status, 0.40 < RC < 0.65 to the presence of a heterozygous deletion, and 1.30 < RC < 1.65 to a heterozygous duplication.

### 2.6. LongPCR Amplification and Gel Electrophoresis

**Figure 2:**
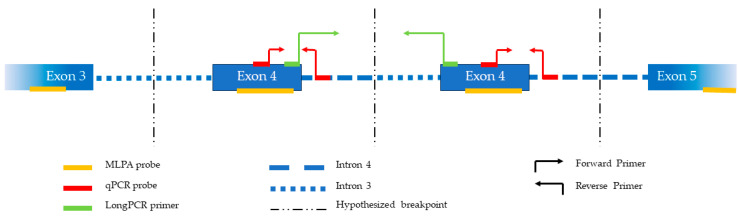
Representation of the duplicated region, probes, and primers used for multiplex ligation-dependent probe amplification (MLPA), quantitative PCR (qPCR) and LongPCR analysis.

To demonstrate that the duplication is in tandem, we used a specific primer pair (primer sequences are reported in Supplementary Materials, Table S1) to amplify the fragment between the end of the first exon 4 and the beginning of the duplicated exon 4 (Figure 2). Therefore, the WT allele was not amplified. PCR amplification was performed for all the patients and a WT control using GoTaq^®^ Long PCR Master Mix (Promega, Madison, WI, USA), according to the manufacturer’s protocol. The amplified sequences were run on a 1.5% agarose gel in tris-acetate-EDTA buffer for 1 h at 120 V. Since the length of the amplified region was unknown, we used both DNA Molecular Weight Marker II (ranging from 23,130 bp to 564 bp) and VIII (ranging from 1114 bp to 67 bp) (Merk, Darmstadt, Germany) as molecular weight references.

## 3. Results

### 3.1. Clinical Report

All the family members are affected by several small and diffuse osteochondromas; none of them have ever undergone surgery to remove OCs.

Pt-1, a 13-year-old girl, at last evaluation presented five OCs at her humerus, radius, femur, and tibia bones. She has pes planus, a lower limb discrepancy of 1 cm, and mild lumbar scoliosis, without any functional limitation. According to the IOR classification [12], she has MO Class IA (no deformities or functional limitations, ≤5 OCs). Quality of life was assessed using EQ-5D-5L, a validated descriptive system of five health-related dimensions: mobility, self-care, usual activities, pain/discomfort, and anxiety/depression. Each area is articulated into five severity levels: no problems, slight problems, moderate problems, severe problems, and extreme problems. The responses are combined in a unique five-digit number ranging from the full health state (‘11111’) to the worst state (‘55555’) [13]. The patient reported a health profile of 11,111 and a health status of 95/100 on the EQ-5D visual analog scale.

Pt-2 is a 41-year-old woman with approximately 16 small and asymptomatic OCs, with no deformities or functional limitations (Class IB). The grandmother, Pt-3, is a 64-year-old woman, with multiple OCs and no deformities or functional limitations (Class IB).

### 3.2. Genetic Results

**Figure 3:**
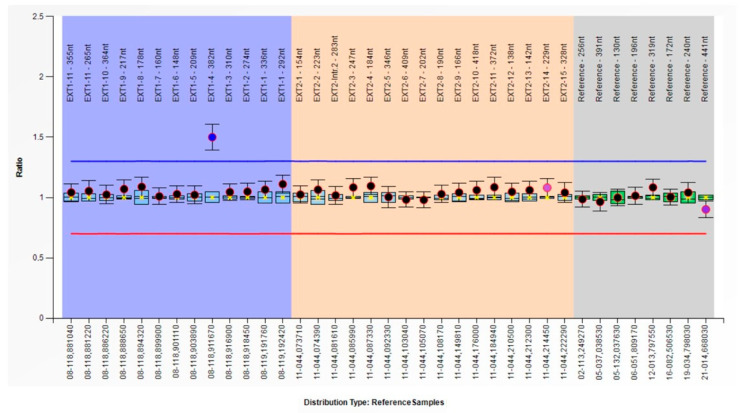
Multiplex ligation-dependent probe amplification (MLPA) results of Pt-1. The dosage quotient (DQ) of the exon 4 probe (L06420) is 1.5, associated to a heterozygous duplication of the target region.

No pathogenic or likely pathogenic point mutation has been detected in *EXT1* and *EXT2* by NGS. The subsequent MLPA analysis allowed us to detect a heterozygous duplication (Figure 3), g.(118847705_118842550)_(118842473_118834776)dup (NG_007455.2), c.(1143_1202)_(1281_1344)dup (NM_000127.2), involving most of exon 4 of *EXT1;* the variant was detected in all the analyzed family components.

In detail, the DQs related to the exon 3, 4, and 5 probes in Pt-1, Pt-2, and Pt-3 are reported in Table 1. The exon 3- and exon 5-related probes have DQ values of 1.01–1.05 and 0.96–1.05, respectively, resulting in a normal copy number status. Differently, the exon 4-related probe has DQ values (1.43–1.54) compatible with heterozygous duplication of the target region. Since that abnormalities detected by a single MLPA probe have a considerable chance of being false positive results, our results required confirmation by a different method. The copy number status of exon 4, also including its adjacent exons, was then evaluated by qPCR. Amplification curves of patients are available for consultation in the Supplementary Materials, Figure S1. Considering this qPCR analysis, the presence of exon 4 duplication has been confirmed in all the family members, with RT values of 1.56, 1.51, and 1.6 (Table 1). As seen in MLPA, both the adjacent exons have a normal copy number status: all the samples show RC values ranging from 1.04 and 1.13 in exon 3, and from 0.93 and 0.99 considering exon 5. Besides confirming what was observed in MLPA, the qPCR analysis also permitted us to extend the length of the duplicated region as follows: g.(118834777_118842440)_(118842551_118847719)dup (NG_007455.2), c.(1128_1202)_(1284+29_1344)dup (NM_000127.2).

**Figure 4:**
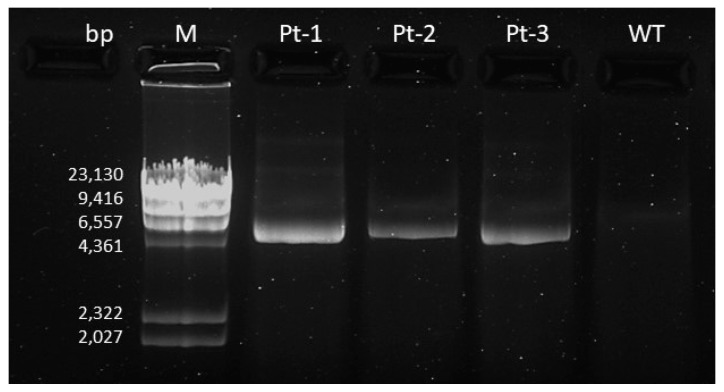
Gel electrophoresis results. bp: base pairs, M: molecular weight marker; WT: wild-type sample; Pt: patient.

According to the ACMG classification criteria, the variant was classified as a variant of unknown significance (VUS, class 3). To demonstrate that the duplication is in tandem and therefore reclassify the duplication, we amplified the fragment between the end of the first exon 4 and the beginning of the duplicated exon 4 (Figure 2). The agarose gel (Figure 4) clearly shows an amplification band for all the patients, with an estimated length between 4361 and 6557 bp, assumed to be the dimension of the duplicated region. As expected, the WT control does not show any amplification band.

The duplication has never been reported before, and according to ACMG guidelines, it has been classified as pathogenic (class 5), based on our experimental data (in-tandem, exon-level duplication, Nonsense-Mediated Decay (NMD) predicted to occur, and segregating in three family members with an *EXT1*-consistent phenotype).

## 4. Discussion

MO is one of the most common skeletal dysplasias, characterized by huge clinical variability. Penetrance is almost complete (96–100%), with only a few cases of incomplete penetrance reported; however, a comprehensive radiologic skeletal survey was not performed in these patients [2]. The clinical severity of the disease depends on the OC site, number, size, and shape [10]. Although in some individuals, the OCs are totally asymptomatic, in most cases, the disease causes bone deformities, pain, and functional limitations, leading to severe progressive disabilities and thus having a significant impact on patients’ quality of life [14]. The diagnosis was based on clinical and radiological findings and was confirmed by a genetic test; nevertheless, up to 30% of cases remain molecularly undiagnosed, possibly because of undetected variants with low-level mosaicism or located within the non-coding region or in a still unknown locus [15]. Identifying the underling genetic variant is crucial to these families since it is a sine qua non to offer prenatal/preimplantation diagnosis and presymptomatic tests for relatives. Moreover, it has been suggested that *EXT1* mutations can be associated with a higher risk for chondrosarcoma; if confirmed, this could allow for different screening protocols if the involved gene is known [16].

Herein, we report a novel heterozygous duplication c.(1128_1202)_(1284+29_1344)dup involving exon 4 of *EXT1* (NM_000127.2), detected in all the affected members of a three-generation Italian family with MO. The duplication is absent from the control population in the Genome Aggregation Database (gnomAD SVs v2.1), and it has not been previously reported in ClinVar, the Leiden Open Variation Database (LOVD), or HGMD. It has been classified as pathogenic according to ACMG criteria since it is an intragenic duplication involving a coding region and it is demonstrated to be in tandem. Moreover, the variant is clearly inherited from the affected grandmother, Pt-3, who had, in turn, inherited the MO disease. To our knowledge, this is the first intragenic pathogenic large duplication described in MO.

All the affected members of the described family have a typical phenotype of MO, at the mild end of the spectrum. In fact, they have OCs at multiple sites, the number of which is on par with the average of the disease population, but they do not have deformities or functional limitations, and Pt-1 reported an overall good quality of life [14]. Patients with *EXT1* duplications, thus, apparently have a clinical presentation coherent with the known intra- and interfamilial heterogeneity of the disease, which can range from almost asymptomatic to severely disabling [12,14]. To date, no genotype–phenotype correlation based on the variant type has been identified, and this report does not suggest otherwise; other factors must be involved in the variable clinical expression [17].

Through our diagnostic experience, we demonstrate the importance of a two-step genetic analysis, composed of NGS sequencing and quantitative techniques, since NGS is not yet capable of detecting every type of CNV, as in our case, and vice versa. Indeed, CNV identification from NGS data, especially in short read sequencing, remains challenging; the detection limit varies depending on several factors, such as read depth, targeted regions, bioinformatic pipeline used, and CNV’s dimension, with the single-exon CNV being more difficult to predict [18,19]. On the other hand, MLPA can also have false negative results, i.e., in the event of CNVs outside the probes’ region (*personal data*). Therefore, both NGS and quantitative approaches should be used in the diagnostic routine in the evaluation of deletion and duplication in *EXT1* and *EXT2* genes in case of clinical suspicion.

In conclusion, this paper extends the mutation spectrum of MO and further underlines the importance of a quantitative analysis in patients with negative sequencing, with a high attention to duplication too, to detect single-exon CNVs that might be underestimated and that could explain some of the molecularly undiagnosed cases.

## Figures and Tables

**Table 1:** Multiplex ligation-dependent probe amplification (MLPA) and quantitative PCR (qPCR) results of exon 3, exon 4, and exon 5. WT: wild-type sample; Pt: patient.

	**MLPA ex.3**	**MLPA ex.4**	**MLPA ex.5**
**Sample**	Dosage Quotation (DQ)		
Pt-1	1.05	1.5	1.02
Pt-2	1.03	1.43	0.96
Pt-3	1.01	1.54	1.05
WT	1.01	0.99	0.98
	**qPCR ex.3**	**qPCR ex.4**	**qPCR ex.5**
**Sample**	Relative Concentration (RC)		
Pt-1	1.06	1.56	0.95
Pt-2	1.04	1.51	0.99
Pt-3	1.13	1.6	0.93
WT	1	1	1

## Data Availability

The identified variant has been added to the Leiden Open Variation Database (LOVD): “https://databases.lovd.nl/shared/genes/EXT1 (accessed on 28 August 2024)”.

## Supplementary Materials

The following supporting information can be downloaded at https://www.mdpi.com/article/10.3390/genes15091169/s1, Figure S1: qPCR amplification curves of patients (left group) and wild-type controls (right group); Table S1: primer sequences.
