# Supplementary material for: A Novel Pathogenic Large Duplication in EXT1 Identified in a Family with Multiple Osteochondromas
Source: Genes (Basel). 2024 Sep 5;15(9):1169. doi: 10.3390/genes15091169 (PMC11430977; doi:10.3390/genes15091169)
Supplement: Supplementary file 1 [file genes-15-01169-s001.zip › genes-3151713-supplementary.pdf]

Target	Forward Primer Sequence (qPCR)	Reverse Primer Sequence (qPCR)
**EXT1 Exon 3**	5′ GGAACCAAGCTGCCGTCATA 3′	3′ TCTGCTGATGTGTTGAAGGCC 5′
**EXT1 Exon 4**	5′ TGTGGGAGGCTTATTTTTCTTCAG 3′	3′ AGCCCAAGAGCCAAGTGGTC 5′
**EXT1 Exon 5**	5′ TTCGTACTACCACAGTATTCATCTTATCTGGG 3′	3′ CCTGGAGGAAATTCACTTACCTAAATTAG 5′
**ACTB**	5′ CAGGTCATCACCATTGGCAAT 3′	3′ CAGTCTCCACTCACCCAGGAAG 5′

Target	Forward Primer Sequence (PCR)	Reverse Primer Sequence (PCR)
**Fragment between duplicated Exons 4**	5′ GAAGATTGTATTAACTACACTAGAG 3′	3′ GAATAGACCTGATTGTAGAAGGA 5′

Table S1. Primer sequences.

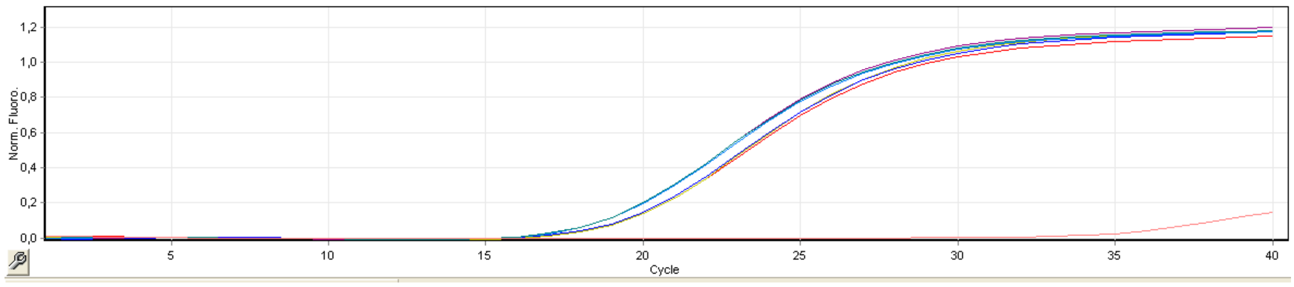

Figure S1. qPCR amplification curves of patients (left group) and wild type controls (right group).
